# Clinical Outcomes of Meniscus Repair with or without Multiple Intra-Articular Injections of Platelet Rich Plasma after Surgery

**DOI:** 10.3390/jcm10122546

**Published:** 2021-06-09

**Authors:** Cheng-Pang Yang, Kung-Tseng Hung, Chun-Jui Weng, Alvin Chao-Yu Chen, Kuo-Yao Hsu, Yi-Sheng Chan

**Affiliations:** 1 Department of Orthopedic Surgery, Division of Sports Medicine Chang Gung Memorial Hospital, College of Medicine, Chang Gung University, Linkou 333, Taiwan; ronnie80097@gmail.com (C.-P.Y.); st900567@gmail.com (K.-T.H.); jim_weng@hotmail.com (C.-J.W.); alvin_ortho@yahoo.com (A.C.-Y.C.); emsequoia@cgmh.org.tw (K.-Y.H.); 2 Bone and Joint Research Center, Chang Gung Memorial Hospital, Linkou 333, Taiwan

**Keywords:** PRP, meniscus repair, meniscectomy, joint injection, osteoarthritis

## Abstract

Preservation of the meniscal volume is crucial in meniscus repair. The goal of this study was to evaluate the clinical outcome of repeated intra-articular platelet-rich plasma (PRP) injections after arthroscopic repair of a traumatic meniscal tear. We retrospectively reviewed 61 primary meniscal repairs in 61 patients (PRP group: 30; non-PRP: 31) from 2017 to 2018. Patients in the PRP group received repeated intra-articular PRP injections in week 2,4,6 after the primary meniscus repair. Subsequent meniscal repair treatment or meniscectomy, knee arthroplasty, and IKDC changes of less than 11.5 points were defined as healing failures. After following up for at least 24 months, the IKDC score was 75.1 ± 13.6, and the Lysholm score was 80.6 ± 14.9 in the PRP group and 72.6 ± 15.8 (IKDC) and 77.7 ± 17.2 (Lysholm) in the non-PRP group. Healing rates of the PRP and the non-PRP groups were 93.3% (Kaplan-Meier 91.6%) and 87.1% (Kaplan-Meier 84.7%), respectively (log rank test *p* = 0.874). Our study is the first to use multiple intra-articular PRP injections to facilitate meniscal healing after meniscal repair. Though selection bias may be present in this study, the PRP group had similar functional outcome and healing rate compared to non-PRP group.

## 1. Introduction

Meniscus tears are commonly diagnosed sports injuries. Normally, treatment options include partial meniscectomy and meniscal repair. Some studies have advocated preserving the meniscus as much as possible [1,2,3,4,5]. In some research, partial meniscectomy is correlated with worse outcomes [6]. However, meniscal tissue is poorly vascularized and has low healing capacity. Some authors have suggested that the failure rate is between 20% and 25% in isolated repair patients [7,8,9,10,11]. At the same time, meniscal repair with anterior cruciate ligament (ACL) reconstruction results in a significantly better healing rate [12,13,14]. One hypothesis is that bone tunnel bleeding may have an important role for meniscal tear healing. This has led to the current focus on biological augmentation, such as platelet-rich plasma (PRP) extracellular matrix or mesenchymal stem cells. PRP contains various growth factors that could theoretically enhance soft tissue healing [15,16,17,18].

In vitro studies have demonstrated improved tenocyte proliferation with use of PRP [19,20]. In the clinical setting, the protocol for PRP augmentation varies greatly. Few authors have compared the outcome of meniscal repair with and without PRP augmentation. Joshua et al. injected PRP mixed with thrombin and calcium chloride intraoperatively, demonstrating a reduced failure rate in isolated meniscal repair, while the protective effect was insignificant in patients who concomitantly received ACL reconstruction [21]. Justin et al. also introduced PRP during arthroscopic surgery and sutures into the meniscal tissue. Their results showed no difference in reoperation rate, functional outcome, or return-to-work rate [22].

Most studies applied PRP a single time with scaffolds intraoperatively. While multiple PRP injections have been used in the management of osteoarthritis [23], no studies have reviewed patients receiving multiple intra-articular PRP injections after meniscal repair. Mihai et al. used intra-articular PRP injection for adolescent grade II meniscus tears. The procedure was only performed once and was found to be effective in improving clinical outcomes [24]. Cook et al. designed an animal model with ACL and meniscal deficiencies. All dogs received leuko-reduced PRP (ACP) or saline at 1, 2, 3, 6, and 8 weeks after insult. The results of the treatment showed significant benefits for ACL repair, range of motion improvement, and pain alleviation [25]. Despite these promising results, injections of PRP alone seemed ineffective in enhancing meniscal healing. Herein, our hypothesis is that multiple intra-articular PRP injections after meniscal repair would promote healing and slow the progression of osteoarthritis. The goal of our study was to compare the clinical outcomes of multiple intra-articular PRP injections after meniscal repair to those of patients who do not receive a PRP injection.

## 2. Materials and Methods

This study was approved by the Biomedical Institutional Review Board of the Chang Gung Hospital. We reviewed a total of 108 primary meniscal repairs on 108 patients at a single institution who received first-time arthroscopic meniscal repair with and without concomitant ACLR by a single surgeon from January 2017 to January 2018. The PRP group included 40 patients, and the non-PRP group initially included 68 cases. All patients underwent complete radiographic analysis, including knee X-rays and MRI, and received clinical evaluations before surgery. Meniscal allograft transplants were excluded. If a patient underwent osteotomy, collateral ligament reconstruction, or PCL reconstruction, or if a patient sustained old femur/tibia fracture or procedures on the contralateral meniscus or contralateral knee, they were excluded. Using these criteria, three patients in the PRP group and 24 in the non-PRP group were excluded. During the follow-up, seven patients in the PRP group and 13 in the non-PRP group were lost to follow-up. A total of 61 primary meniscal repairs on 61 patients (PRP group: 30; non-PRP: 31) were ultimately included.

All surgery was performed under general anesthesia using standard procedures (rasping, reduction, and fixation). The all-inside technique with a FastFix device (Smith and Nephew, Cordova, TN, USA) was primarily used. If patients had a bucket-handle tear extending from the posterior horn middle body, an inside-out suture was applied first for better reduction. Additional outside-in sutures may have been placed for middle body or anterior horn repair. Demographic data is listed in Table 1. The tear pattern, surgical technique, and other intraoperative finding are listed in Table 2. Pure bucket-handle tears were categorized into longitudinal tear patterns. The use of PRP after meniscal repair was primarily based on the decision of the senior surgeons rather than specific criteria. Complex tear patterns, large defects, concomitant cartilage injuries, and long durations before surgery were the primary reasons for using PRP injections.

For the PRP injection protocol, we arranged the intra-articular injection at the outpatient clinic 2, 4, and 6 weeks postoperatively. Thirty patients received one course (three doses) of PRP injection after meniscal repair. Among these, 17 underwent concomitant ACL reconstruction (13 isolated meniscal repairs) at the index surgery. In the other group, 31 patients received meniscal repair (17 patients with ACLR and 14 patients with isolated meniscal repair) without subsequent PRP injection. All patients had follow-up for more than 24 months (average 33.0 ± 6.9 months).

### 2.1. Platelet-Rich Plasma Augmentation

PRP injection was performed using the Regen Kit (REGENLAB, RegenACR-C Classic, Le Mont-sur-Lausanne, Switzerland). The Regen Kit is a fully enclosed system that maintains sterility throughout the entire process and uses a dual spin system. To prepare PRP with concentrations four to six times the average of normal values, 10 mL of blood was first drawn from the patient’s upper limb cubital vein using an 18 G needle; subsequently, 5 mL of acid citrate dextrose solution-A was added to the sample as an anticoagulant. Local anesthetic agent was not injected. The patient was placed in a supine position with the knee at 90 degrees of flexion. The skin of the injection site was prepared and draped, and liquid PRP was injected under sterile conditions using a 23 G needle through the anterolateral portal. Limited movement was allowed for 24 h. Resting or ice packing for 3 days was recommended in cases of pain or swelling. Patients did not take nonsteroidal anti-inflammatory drugs (NSAIDs) for two weeks before the next course of PRP injection.

### 2.2. Outcome Measurement

Mean follow-up time for both the PRP-augmented and non-PRP groups was 39.0 ± 6.9 months (range, 24.1–44.6 months). Functional outcomes were assessed preoperatively and at 24 months postoperatively with the International Knee Documentation Committee (IKDC) score and Lysholm score. An IKDC score change of less than 11.5 points was categorized as a failed surgery according to a previous study [26]. We also compared pain scores before and 1, 3, 6, and 12 months after the operation. Clinical outcome data were also collected and were available for 100% of patients in the non-PRP and PRP groups.

### 2.3. Radiographic Evaluation

As the meniscus serves as the shock absorber in the knee joint, an intact structure protects the hyaline cartilage and avoids early osteoarthritic changes. We compared standing knee X-rays preoperatively and postoperatively. We included the alignment measurement (tibiofemoral angle, joint line congruency angle) and Kellgren-Lawrence grade. Radiographic evaluation was performed and rechecked by three authors of this study (CP Yang, KT Hung, and CJ Weng). The radiographic measurements are listed in Table 3.

#### Statistical Analysis

We used the Statistical Package for the Social Sciences (IBM SPSS Statistics 23.0) (IBM Inc., Armonk, NY, USA) and Microsoft Office Excel (Microsoft Office 2016). All categorical data were analyzed using Fisher’s exact test. The VAS score, IKDC and Lysholm score were analyzed using the two-tailed Mann–Whitney U test or unpaired *t*-test. Results were considered statistically significant at a *p* value < 0.05.

## 3. Results

### 3.1. Descriptive Statistics

There were 44 men (72%) and 17 women in this study with a mean age of 36.4 ± 12.3 years old and BMI of 25.2 ± 3.2 kg/m^2^. In the PRP group, there were 25 men and 5 women with a mean age of 37.3 ± 11.2 years. In the non-PRP group, there were 19 men and 12 women with a mean age of 35.6 ± 13.4 years. The two groups showed no significant differences. The tear pattern, ACL status, suture technique, and knee joint alignment were not significantly different between the PRP-treated and non-PRP-treated groups. However, the average suture number was 3.9 ± 1.6 in the PRP group and 2.2 ± 1.1 in the non-PRP group (*p* < 0.001). This was compatible with the senior surgeon’s criteria that patients with more complex tear patterns, large tear sizes, and devascularized tissue were indicated for PRP injection. All demographic data are listed in Table 1. The tear pattern, surgical technique, and other intraoperative finding are listed in Table 2.

### 3.2. Functional Outcome and Meniscus Healing Failure

Preoperative IKDC and Lysholm scores were not different between the two groups. In the postoperative follow-up, the IKDC score was 75.1 ± 13.6, and the Lysholm score was 80.6 ± 14.9 in the PRP group. Changes after surgery and PRP injection were 25.5 ± 10.4 in the IKDC group and 27.8 ± 11.7 in the Lysholm group. In the non-PRP group, the postoperative IKDC score was 72.6 ± 15.8, and the Lysholm score was 77.7 ± 17.2. Changes after surgery without PRP were 22.7 ± 10.0 for IKDC and 24.4 ± 11.1 for Lysholm. Postoperative functional score and changes in these scores were not different between the two groups. The functional outcome data are listed in Table 4.

**Figure 1:**
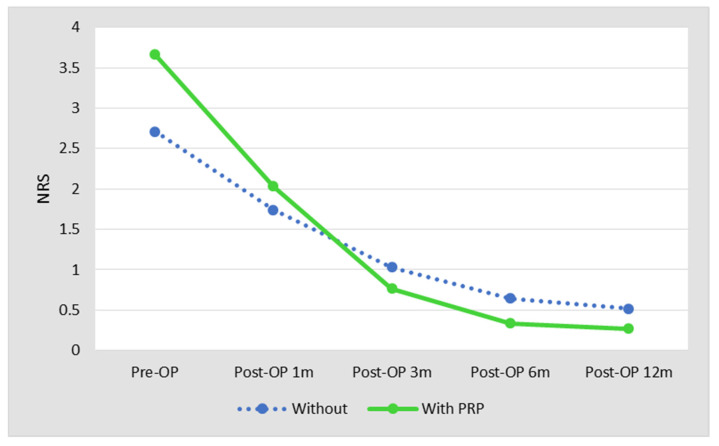
Patients in the PRP group had higher NRS pain scores than those in the non PRP group preoperatively. After the operation, the pain score showed no difference between two groups.

Patients in the PRP group had higher NRS pain scores than those in the non PRP group preoperatively (PRP: 3.7 ± 1.1 (2–6); non-PRP: 2.7 ± 0.8 (1–5), *p*< 0.001). Pain scores were determined at 1, 3, 6, and 12 months postoperatively and were not different. The pain score comparison is shown in Figure 1.

As mentioned above, we defined meniscal healing failure as subsequent meniscal repair or meniscectomy, or subsequent total knee arthroplasty. In addition, IKDC change scores of less than 11.5 points were categorized as failed operations. Until the last follow-up, only one patient in the non-PRP group received revision meniscus repair. However, two patients in the PRP group and another three patients in the non-PRP group had a change in IKDC of less than 11.5. Using this definition, healing rates of the PRP and non-PRP groups were 93.3% (Kaplan-Meier 91.6%) and 87.1% (Kaplan-Meier 84.7%), respectively (log rank test *p* = 0.874). The description of all failure cases is listed in Table 5.

### 3.3. Radiographic Result

In the preoperative knee AP view, neither alignment measurement nor Kellgren-Lawrence grading were significantly different. The X-ray was rechecked after 2 years of follow-up. Similar to the preoperative condition, the X-ray at the last follow-up also exhibited no difference in the alignment measurement and Kellgren Lawrence grading. Radiographic results are shown in Table 4. We observed that three patients in the PRP group and one patient in the non-PRP group had improved KL grades at the last follow-up. However, as the Kellgren Lawrence grade only focuses on the Knee AP view, we were unsure whether these changes were due to poor X-ray quality.

## 4. Discussion

The meniscus plays an important role in the knee joint, as it plays a role in shock absorption and transmission, joint stabilization, proprioception, lubrication and nutrition of the articular cartilage [27]. Biomechanical studies have shown that a loss of meniscal integrity leads to changes in kinematics and loading of the knee joint. Even a loss of only 15–34% of the meniscus tissue increases the load on the hyaline cartilage by up to 350% [28]. Vascularization and nutritional status of the injured meniscus area, as well as the type of meniscus tear, are important indicators for the success of meniscus reconstruction. The inner 2/3 of the meniscus (“white-white”) is nourished by diffusion of factors from the synovial fluid, while the peripheral “red-red zone” has a vascular supply. Between the white-white zone and the red-red zone is a red-white transition zone. Due to its avascular nature, meniscal healing is a critical issue after injury. In the primary meniscal repair setting, some studies regarding isolated repair in ligament-stable knees observed variable clinical healing or success rates ranging between 33% and 76% [8,10,11]. As many researchers suggest, concomitant ACL reconstruction surgery may improve the healing rates of a repaired meniscus compared to isolated repair [9,12]. Research has focused on promoting healing with external stimulants, such as fibrin clots, fibrin glue, synovial grafts, periosteum and mesenchymal stem cells [29,30,31,32,33]. PRP has been widely used in sports medicine with a variety of properties and applied methods.

This is the first study using multiple intra-articular PRP injections in meniscal repair. The results showed significant improvement compared to preoperative status in both the PRP injection and control groups (ΔIKDC score: PRP group: 25.5 ± 10.4; non-PRP group: 22.7 ± 10.0, *p* = 0.291/ΔLysholm score: PRP group: 27.8 ± 11.7; non-PRP group: 24.4 ± 11.1, *p* = 0.253). The postoperative function scores and improvement in the PRP group showed no significant difference compared to non-PRP group. In addition to functional outcome, we also examined the failure rate of meniscal healing. Most previous studies used clinical signs, such as subsequent meniscectomy, revision meniscal repair, or subsequent total/partial knee arthroplasty, to determine procedure success. Kaminski R et al. defined treatment failure as no visible healing during a second-look arthroscopy or less than 50% healing of the tear width versus unstable repair on magnetic resonance imaging review [34].

MRI is the most accurate tool for the evaluating meniscal injury [35]. However, we did not repeat the MRI, as a previous study did not support using MRI for the detection of meniscal healing. Nicolas et al. identified some abnormal hypersignals presented on MRI examination ten years after meniscal repair [36]. Conversely, we used change in IKDC score as a criterion of successful healing. We used the criteria proposed by James et al. in 2006, suggesting that a change in score of 11.5 points had the highest sensitivity, and a change in score of 20.5 points had the highest specificity to define a successful improvement in knee function [26]. Using these definitions, although only one patient received revision meniscus repair, two patients in the PRP group and four in the non-PRP group were considered to have failed healing. The healing rate was 93.3% (Kaplan-Meier 91.6%) in the PRP group and 87.1% (Kaplan-Meier 84.7%) in the non-PRP group (Log rank test *p* = 0.874).

In research published by Justin el al., 35 isolated arthroscopic meniscus repairs from between 2008 and 2011 were assessed [22], 15 (43%) of which were augmented with PRP, and 20 (57%) of which were performed without PRP augmentation. PRP was sutured into the repair site during the setting of an inside-out repair. In their results, the reoperation rate was not different between the PRP (27% (4 of 15)) and non-PRP group (25% (5 of 20); *p* = 0.89). Functional outcome measures were not different between the two groups (mean IKDC score, 69; with PRP and 76 without PRP; *p* = 0.288; mean, Tegner Lysholm Knee Scoring Scale, 66 with PRP and 89 without PRP; *p* = 0.065). A recent study published by Joshua el al. reviewed 550 patients (28.8 +/− 11.2 years) who received meniscal repair surgery with PRP (*n* = 203 total) or without PRP (*n* = 347) and with (*n* = 399) or without (*n* = 151) concurrent ACL reconstruction [21]. The 3-year survival rate was 17.0% of patients without PRP and 14.6% of patients with PRP (*p* = 0.60). Considering isolated meniscal repairs (20.3% failures at 3 years), PRP was independently associated with a reduced risk of failure. Among meniscal repairs with concomitant ACL reconstruction (14.1% failures at 3 years), PRP was not independently associated with risk of failure, and there was no difference between PRP preparation systems (*p* = 0.78). In our study, we included patients with both isolated meniscal repair and repair with concomitant ACL reconstruction because traumatic meniscus injury is commonly detected with ligament injuries, especially ACL tears. The incidence of traumatic meniscus tears in patients with an injured ACL ranged between 57% and 96%. We considered that inclusion of these patients would make the model a more approximate clinical scenario. In addition, the number of concomitant ACL reconstructions was not different between the two groups (PRP: 50%, non-PRP: 54.8%, *p* = 0.866) in our study. As a result, the current study findings may help further define the role of multiple PRPs in knee meniscal repair.

Selection of patients receiving PRP injections has varied in different studies. The abovementioned series of Joshua et al. is the largest study on PRP augmentation in meniscal repair to date. They had a total of 550 patients, with 203 treated with PRP and 347 without PRP. Selection of patients receiving PRP was based on the year of surgery. They did not use PRP before 2010 and started to apply this procedure from January 2010 to February 2015. Demographic results showed a significant difference only in tear pattern, with a larger proportion of vertically oriented tears in the non-PRP group compared to the PRP group (96% vs. 73%, *p* < 0.001) [21]. In the study performed by Justin et al., surgery was performed by three different surgeons; two used PRP augmentation on their patients, while the other did not. The selection criteria were not mentioned in their study. The author admitted that it was uncertain as to whether PRP was used on more difficult tears. The demographic data of their study also exhibited significant differences, including BMI and age, which may affect collagen and cartilage degeneration of the knee [22]. In our study, the patients in both groups were not randomized, as the senior surgeon was likely to select a more complex tear pattern and poorer vascularity for PRP augmentation. Although the demographics showed no difference between the two groups, there might be some selection bias in that the PRP group had less healing potential. However, the functional outcomes and failure rate of the two groups were not different. Kaminski et al. published the first double-blind, placebo-controlled study comparing PRP augmentation and non PRP patients after repair of unstable, complete vertical meniscus tears [34]. They examined 17 patients in the PRP group and 18 in the non-PRP group. PRP or placebo was introduced to the tear site intraoperatively. They re-examined the patients after 18 weeks. The meniscal healing rate was significantly higher in the PRP-treated group than in the control group (85% vs. 47%, *p* = 0.048). In addition, the IKDC score, WOMAC score, and KOOS were also significantly better in the PRP-treated group. These previous studies and our study may provide a glimpse of the effect of PRP.

While most research used PRP augmentation intraoperatively with fibrin or other scaffolds directly at the tear site, we applied intra-articular injection in the outpatient department for a number of reasons. In a previous animal study, the repaired meniscus showed a synovial pannus at 2 weeks and a gradual increase in strength until 12 weeks. Cell lineage is mostly fibroblastic during the first 6 weeks [37,38]. We hope to facilitate meniscal healing in this stage. This stage relies on a variety of growth factors with chemotactic and mitogenic functions that stimulate further cellular and vascular proliferation [39]. Kobuna et al. studied microangiography in dogs and found that vessels located on the femoral surface and the inner part of the meniscus reached the sutured area. At 6 weeks, lesions exhibited healing with fibrovascular tissue [40]. We hypothesized that a repeated injection of PRP in the first 6 weeks would enhance vascularity and prevent nonhealing. As the procedure can be easily and regularly performed in outpatient clinics, we chose the intra-articular method rather than direct administration at the tear site. The other reason for selecting this method was because we worried that the PRP clot would be lost with fluid pumping or leak from the tear cleft during repair. We believe that the intra-articular method is sufficient to supply growth factors to the injured site. Mihai et al. used intra-articular injection for isolated grade II meniscus tears in adolescent patients without surgical intervention [24]. There were a total of 30 patients with a mean age of 13.93 years old. Their protocol contained only one dose of PRP injection, and patients were followed up for 3 months. The mean value before injection on the numerical rating scale (NRS) of pain was 7.73, while the number after treatment was 2.0. After treatment, 76.7% of patients had “excellent” and “good” outcomes, while before injection, only 3% of the patients had a “good” score. In an animal study published by James et al., 12 dogs received partial ACL transection and meniscal release in one knee [25]. At 1, 2, 3, 6, and 8 weeks after surgery, dogs were administered intra-articular injections (2 mL) of either leuko-reduced PRP (*n* = 6) or saline (*n* = 6, control group). The comfortable range of motion (CROM), pain, effusion, kinetics, and radiographic, arthroscopic and histological assessments were assessed at the 6-month endpoint. The control group exhibited significantly (*p* < 0.04) more CROM loss and pain (*p* < 0.01) in the affected hind limbs than PRP-treated dogs. Arthroscopically, saline-treated knees showed moderate to severe synovitis, further ACL disruption, and medial compartment cartilage loss, while PRP-treated knees showed evidence of ACL repair and less severe synovitis. However, meniscal healing was not affected by PRP injection, nor did it slow the development or progression of OA compared to controls. In contrast to our study, this animal model treated meniscal repair with only PRP injection without repair. We believe that prior to biological augmentation, strong and stable repaired tissue is the primary determinant for meniscal healing. However, the positive effect of multiple intra-articular injections into the poorly vascularized ACL in this study still gives us more confidence in this augmentation method after meniscal repair.

There are some limitations to our study. First, this was a retrospective study, and patients were not randomly selected. In addition, selection of PRP injection was based on the senior surgeon’s criteria. We believe that the healing potential in this group was lower. However, the functional result and failure rate showed a trend that was better than that of the non-PRP group. Second, the mean follow-up time was only 2 years. To evaluate the protective effect of the healthy meniscus of cartilage, longer follow-up for the detection of early osteoarthritis changes is necessary. On the other hand, we thought 24 months was enough for the evaluation of clinical function and early failure.

## 5. Conclusions

Ours is the first clinical study to use multiple intra-articular PRP injections to facilitate meniscal healing after meniscal repair. However, selection bias may be present in this study. After a follow-up of more than 2 years, the PRP group had similar functional outcome and healing rate compared to non-PRP group. A prospective, randomized control study with long-term follow-up is needed in the future to confirm the benefits of this application.

## Figures and Tables

**Table 1:** Demographic data.

	All Patients (*n* = 61)	with PRP (*n* = 30)	without PRP (*n* = 31)	*p* Value
Demographics				
Male	44	25	19	0.055
Female	17	5	12	
Age, yr, mean ± SD	36.4 ± 12.3	37.3 ± 11.2	35.6 ± 13.4	0.610
Body mass index, kg/m^2^, mean ± SD	25.2 ± 3.2	25.7 ± 3.2	24.8 ± 3.3	0.307
Smoking	6	3	3	1.000 **
Injury to surgery, wk, mean ± SD (range)	81.5 ± 145.8	105.6 ± 169.9	58.3 ± 116.1	0.286 *
	(1.0–579.4)	(2.4–579.4)	(1.0–521.9)	
Follow-up time, mo, mean ± SD (range)	33.0 ± 6.9	28.9 ± 5.9	38.9 ± 5.4	<0.001 *
	(24.1–44.6)	(25.2–38.3)	(24.1–44.6)	

PRP, platelet-rich plasma; SD, standard deviation; * Mann Whitney U test was applied for data not following normal distribution. ** Fisher’s exact test was applied.

**Table 2:** Intraoperative status.

	All Patients (*n* = 61)	with PRP (*n* = 30)	without PRP (*n* = 31)	*p* Value
Intraoperative medial cartilage grading				
Grade 0	43	18	25	0.137 **
Grade 1	5	4	1	
Grade 2	7	5	2	
Grade 3	4	1	3	
Grade 4	2	2	0	
Intraoperative lateral cartilage grading				
Grade 0	50	26	24	0.222 **
Grade 1	2	1	1	
Grade 2	5	3	2	
Grade 3	4	0	4	
Grade 4	0	0	0	
Meniscus condition				
Discoid meniscus	8	4	4	1.000 **
Meniscus side				
Medial	15	11	4	0.077
Lateral	34	13	21	
Both	12	6	6	
Tear pattern				
Longitudinal	29	16	13	0.337 **
Horizontal	9	5	4	
Radial	4	0	4	
Complex	16	8	8	
Root	3	1	2	
ACL status				
Concomitant ACL reconstruction	34	17	17	0.866
Repair technique				
All-inside	42	15	27	0.005 **
All-inside + Inside-out	11	9	2	
All-inside + Outside-in	5	3	2	
All-inside + Inside-out + Outside-in	3	3	0	
Total number of sutures, mean ± SD	3.0 ± 1.6	3.9 ± 1.6	2.2 ± 1.1	<0.001

PRP, platelet-rich plasma; SD, standard deviation; ACL, anterior cruciate ligament; ** Fisher’s exact test was applied.

**Table 3:** Comparison of radiographic finding.

	All Patients (*n* = 61)	with PRP (*n* = 30)	without PRP (*n* = 31)	*p* Value
Preoperative radiographic measurement				
Tibiofemoral angle, degrees, mean ± SD	3.9 ± 2.6	3.6 ± 3.0	4.2 ± 2.3	0.342
Joint line congruency angle, degrees, mean ± SD	1.8 ± 1.0	2.0 ± 1.2	1.5 ± 0.8	0.057
Postoperative radiographic measurement				
Tibiofemoral angle, degrees, mean ± SD	4.2 ± 2.1	3.8 ± 3.0	4.7 ± 2.1	0.111
Joint line congruency angle, degrees, mean ± SD	1.3 ± 0.8	1.5 ± 0.7	1.1 ± 0.9	0.122
Preoperative knee Kellgren-Lawrence grading				
Grade 0	10	5	5	0.353 **
Grade 1	19	12	7	
Grade 2	29	11	18	
Grade 3	3	2	1	
Grade 4	0	0	0	
Postoperative knee Kellgren-Lawrence grading				
Grade 0	10	5	5	0.290 **
Grade 1	22	14	8	
Grade 2	27	10	17	
Grade 3	2	1	1	
Grade 4	0	0	0	

PRP, platelet-rich plasma; SD, standard deviation; ACL, anterior cruciate ligament; ** Fisher’s exact test was applied.

**Table 4:** Functional outcome.

	All Patients (*n* = 61)	with PRP (*n* = 30)	with PRP Group *p* Value	without PRP (*n* = 31)	without PRP Group *p* Value	between Group *p* Value
Preoperative IKDC score	49.7 ± 9.4	49.6 ± 9.5	<0.001	49.8 ± 9.5	<0.001	0.941
	(29.9–72.4)	(32.2–72.4)		(29.9–66.7)		
Postoperative IKDC score	73.8 ± 14.7	75.1 ± 13.6		72.6 ± 15.8		0.593 *
	(34.5–95.4)	(34.5–93.1)		(39.1–95.4)		
Δ IKDC score	24.0 ± 10.3	25.5 ± 10.4		22.7 ± 10.0		0.291
	(2.3–50.6)	(2.3–50.6)		(5.7–43.7)		
Preoperative Lysholm score	53.0 ± 14.0	52.8 ± 12.9	<0.001	53.3 ± 15.2	<0.001	0.900
	(20–80)	(29–80)		(20–78)		
Postoperative Lysholm score	79.2 ± 16.0	80.6 ± 14.9		77.7 ± 17.2		0.670 *
	(33–100)	(50–100)		(33–99)		
Δ Lysholm score	26.1 ± 11.5	27.8 ± 11.7		24.4 ± 11.1		0.253
	(7–49)	(10–48)		(7–49)		

PRP, platelet-rich plasma; SD, standard deviation; IKDC, International Knee Documentation Committee. Δ scores were calculated by subtracting the preoperative score from the final score. * Mann Whitney U test was applied for data not following normal distribution.

**Table 5:** Failure case characteristics.

PRP	Sex	Age (yr)	Side	Tear Pattern	ACL Repair	Repair Technique	Number of Sutures	Δ IKDC Score	Δ Lysholm Score	Failure Time (mo)	Revision Meniscal Repair
Y	M	35.0	Both	Longitudinal	Y	All-inside + Inside-out	5	6.9	13	21.0	N
Y	M	44.7	Both	Longitudinal	Y	All-inside	2	2.3	16	24.9	N
N	M	48.2	Lateral	Root tear	Y	All-inside	1	5.7	9	37.4	N
N	M	18.8	Medial	Longitudinal	N	All-inside	1	9.2	13	33.1	N
N	F	46.1	Lateral	Longitudinal	N	All-inside	2	6.9	16	18.6	N
N	M	26.1	Lateral	Longitudinal	Y	All-inside	1	6.9	10	21.2	Y

PRP, platelet-rich plasma; ACL, anterior cruciate ligament; IKDC, International Knee Documentation Committee. Failure: Change of IKDC score < 11.5 and/or underwent revision meniscus repair.

## Data Availability

The dataset supporting the conclusions of this article is available from the corresponding author on reasonable request.

## References

[1] Persson F., Turkiewicz A., Bergkvist D., Neuman P., Englund M. (2018). The risk of symptomatic knee osteoarthritis after arthroscopic meniscus repair vs partial meniscectomy vs. the general population. Osteoarthr. Cartil..

[2] Englund M., Lohmander S. (2004). Risk factors for symptomatic knee osteoarthritis fifteen to twenty-two years after meniscectomy. Arthritis Rheum..

[3] Faucett S.C., Geisler B., Chahla J., Krych A.J., Kurzweil P.R., Garner A.M., Liu S., Laprade R.F., Pietzsch J.B. (2019). Meniscus Root Repair vs Meniscectomy or Nonoperative Management to Prevent Knee Osteoarthritis After Medial Meniscus Root Tears: Clinical and Economic Effectiveness. Am. J. Sports Med..

[4] McDermott I.D., Amis A.A. (2006). The consequences of meniscectomy. J. Bone Jt. Surg. Br. Vol..

[5] Petty C.A., Lubowitz J.H. (2011). Does Arthroscopic Partial Meniscectomy Result in Knee Osteoarthritis? A Systematic Review with a Minimum of 8 Years’ Follow-up. Arthrosc. J. Arthrosc. Relat. Surg..

[6] Papalia R., Del Buono A., Osti L., Denaro V., Maffulli N. (2011). Meniscectomy as a risk factor for knee osteoarthritis: A systematic review. Br. Med. Bull..

[7] Nepple J.J., Dunn W.R., Wright R.W. (2012). Meniscal Repair Outcomes at Greater Than Five Years: A systematic literature review and meta-analysis. J. Bone Jt. Surg. Am. Vol..

[8] Fillingham Y.A., Riboh J.C., Erickson B.J., Bach J.B.R., Yanke A.B. (2017). Inside-Out Versus All-Inside Repair of Isolated Meniscal Tears: An Updated Systematic Review. Am. J. Sports Med..

[9] Westermann R.W., Wright R.W., Spindler K.P., Huston L., Wolf B.R., Cox C.L., Kaeding C.C., Flanigan D.C., Magnussen R.A., Matava M.J. (2014). Meniscal Repair With Concurrent Anterior Cruciate Ligament Reconstruction: Operative success and patient outcomes at 6-year follow-up. Am. J. Sports Med..

[10] Nakayama H., Kanto R., Kambara S., Kurosaka K., Onishi S., Yoshiya S., Yamaguchi M. (2017). Clinical outcome of meniscus repair for isolated meniscus tear in athletes. Asia-Pacific J. Sports Med. Arthrosc. Rehabil. Technol..

[11] Grant J.A., Wilde J., Miller B.S., Bedi A. (2012). Comparison of Inside-Out and All-Inside Techniques for the Repair of Isolated Meniscal Tears: A systematic review. Am. J. Sports Med..

[12] Korpershoek J.V., De Windt T.S., Vonk L.A., Krych A.J., Saris D.B. (2020). Does Anterior Cruciate Ligament Reconstruction Protect the Meniscus and Its Repair? A Systematic Review. Orthop. J. Sports Med..

[13] Rodríguez-Roiz J.M., Sastre S., Popescu D., Montañana-Burillo J., Combalia-Aleu A. (2020). The relationship between ACL reconstruction and meniscal repair: Quality of life, sports return, and meniscal failure rate—2- to 12-year follow-up. J. Orthop. Surg. Res..

[14] Pathak S., Bharadwaj A., Patil P., Raut S., Rv S. (2020). Functional Outcomes of Arthroscopic Combined Anterior Cruciate Ligament Reconstruction and Meniscal Repair: A Retrospective Analysis. Arthrosc. Sports Med. Rehabil..

[15] Halpern B.C., Chaudhury S., Rodeo S.A. (2012). The Role of Platelet-Rich Plasma in Inducing Musculoskeletal Tissue Healing. HSS J..

[16] Metcalf K.B., Mandelbaum B.R., McIlwraith C.W. (2013). Application of Platelet-Rich Plasma to Disorders of the Knee Joint. Cartilage.

[17] Paoloni J., De Vos R.J., Hamilton B., Murrell G.A.C., Orchard J. (2011). Platelet-Rich Plasma Treatment for Ligament and Tendon Injuries. Clin. J. Sport Med..

[18] Grambart S.T. (2015). Sports Medicine and Platelet-rich Plasma: Nonsurgical therapy. Clin. Podiatr. Med. Surg..

[19] Braun H.J., Wasterlain A.S., Dragoo J.L. (2013). The Use of PRP in Ligament and Meniscal Healing. Sports Med. Arthrosc. Rev..

[20] Kaleka C.C., Debieux P., da Costa Astur D., Arliani G.G., Cohen M. (2014). Updates in biological therapies for knee injuries: Menisci. Curr. Rev. Musculoskelet. Med..

[21] Everhart J., Cavendish P.A., Eikenberry A., Magnussen R.A., Kaeding C.C., Flanigan D.C. (2019). Platelet-Rich Plasma Reduces Failure Risk for Isolated Meniscal Repairs but Provides No Benefit for Meniscal Repairs With Anterior Cruciate Ligament Reconstruction. Am. J. Sports Med..

[22] Griffin J.W., Hadeed M.M., Werner B., Diduch D.R., Carson E.W., Miller M.D. (2015). Platelet-rich Plasma in Meniscal Repair: Does Augmentation Improve Surgical Outcomes?. Clin. Orthop. Relat. Res..

[23] Filardo G., Kon E., Roffi A., Di Matteo B., Merli M.L., Marcacci M. (2015). Platelet-rich plasma: Why intra-articular? A systematic review of preclinical studies and clinical evidence on PRP for joint degeneration. Knee Surg. Sports Traumatol. Arthrosc..

[24] Popescu M.B., Carp M., Tevanov I., Nahoi C.A., Stratila M.A., Haram O.M., Ulici A. (2020). Isolated Meniscus Tears in Adolescent Patients Treated with Platelet-Rich Plasma Intra-articular Injections: 3-Month Clinical Outcome. BioMed Res. Int..

[25] Cook J.L., Smith P.A., Bozynski C.C., Kuroki K., Cook C.R., Stoker A.M., Pfeiffer F.M. (2016). Multiple injections of leukoreduced platelet rich plasma reduce pain and functional impairment in a canine model of ACL and meniscal deficiency. J. Orthop. Res..

[26] Irrgang J.J., Anderson A.F., Boland A.L., Harner C.D., Neyret P., Richmond J.C., Shelbourne K.D., International Knee Documentation Committee (2006). Responsiveness of the International Knee Documentation Committee Subjective Knee Form. Am. J. Sports Med..

[27] Makris E.A., Hadidi P., Athanasiou K.A. (2011). The knee meniscus: Structure–function, pathophysiology, current repair techniques, and prospects for regeneration. Biomaterials.

[28] Radin E.L., De Lamotte F., Maquet P. (1984). Role of the menisci in the distribution of stress in the knee. Clin. Orthop. Relat. Res..

[29] Guo W., Xu W., Wang Z., Chen M., Hao C., Zheng X., Huang J., Sui X., Yuan Z., Zhang Y. (2018). Cell-Free Strategies for Repair and Regeneration of Meniscus Injuries through the Recruitment of Endogenous Stem/Progenitor Cells. Stem Cells Int..

[30] Kawanishi Y., Nakasa T., Shoji T., Hamanishi M., Shimizu R., Kamei N., Usman M.A., Ochi M. (2014). Intra-articular injection of synthetic microRNA-210 accelerates avascular meniscal healing in rat medial meniscal injured model. Arthritis Res. Ther..

[31] Chen M., Guo W., Gao S., Hao C., Shen S., Zhang Z., Wang Z., Wang Z., Li X., Jing X. (2018). Biochemical Stimulus-Based Strategies for Meniscus Tissue Engineering and Regeneration. BioMed Res. Int..

[32] Guo W., Liu S., Zhu Y., Yu C., Lu S., Yuan M., Gao Y., Huang J., Yuan Z., Peng J. (2015). Advances and Prospects in Tissue-Engineered Meniscal Scaffolds for Meniscus Regeneration. Stem Cells Int..

[33] Niu W., Guo W., Han S., Zhu Y., Liu S., Guo Q. (2016). Cell-Based Strategies for Meniscus Tissue Engineering. Stem Cells Int..

[34] Kaminski R., Kulinski K., Kozar-Kaminska K., Wielgus M., Langner M., Wasko M.K., Kowalczewski J., Pomianowski S. (2018). A Prospective, Randomized, Double-Blind, Parallel-Group, Placebo-Controlled Study Evaluating Meniscal Healing, Clinical Outcomes, and Safety in Patients Undergoing Meniscal Repair of Unstable, Complete Vertical Meniscal Tears (Bucket Handle) Augmented with Platelet-Rich Plasma. BioMed Res. Int..

[35] Lefevre N., Naouri J.F., Herman S., Gerometta A., Klouche S., Bohu Y. (2016). A Current Review of the Meniscus Imaging: Proposition of a Useful Tool for Its Radiologic Analysis. Radiol. Res. Pr..

[36] Pujol N., Tardy N., Boisrenoult P., Beaufils P. (2013). Magnetic Resonance Imaging is not suitable for interpretation of meniscal status ten years after arthroscopic repair. Int. Orthop..

[37] Kawai Y., Fukubayashi T., Nishino J. (1989). Meniscal suture. An experimental study in the dog. Clin. Orthop. Relat. Res..

[38] Webber R.J., York J.L., Vanderschilden J.L., Hough A.J. (1989). An organ culture model for assaying wound repair of the fibrocartilaginous knee joint meniscus. Am. J. Sports Med..

[39] Werner S., Grose R. (2003). Regulation of Wound Healing by Growth Factors and Cytokines. Physiol. Rev..

[40] Kobuna Y., Shirakura K., Niijima M. (1995). Meniscal repair using a flap of synovium. An experimental study in the dog. Am. J. Knee Surg..

